# Reduced Distractibility in a Remote Culture

**DOI:** 10.1371/journal.pone.0026337

**Published:** 2011-10-19

**Authors:** Jan W. de Fockert, Serge Caparos, Karina J. Linnell, Jules Davidoff

**Affiliations:** Department of Psychology, Goldsmiths, University of London, London, United Kingdom; University of Manchester, United Kingdom

## Abstract

**Background:**

In visual processing, there are marked cultural differences in the tendency to adopt either a global or local processing style. A remote culture (the Himba) has recently been reported to have a greater local bias in visual processing than Westerners. Here we give the first evidence that a greater, and remarkable, attentional selectivity provides the basis for this local bias.

**Methodology/Principal Findings:**

In Experiment 1, Eriksen-type flanker interference was measured in the Himba and in Western controls. In both groups, responses to the direction of a task-relevant target arrow were affected by the compatibility of task-irrelevant distractor arrows. However, the Himba showed a marked reduction in overall flanker interference compared to Westerners. The smaller interference effect in the Himba occurred despite their overall slower performance than Westerners, and was evident even at a low level of perceptual load of the displays. In Experiment 2, the attentional selectivity of the Himba was further demonstrated by showing that their attention was not even captured by a moving singleton distractor.

**Conclusions/Significance:**

We argue that the reduced distractibility in the Himba is clearly consistent with their tendency to prioritize the analysis of local details in visual processing.

## Introduction

Variation in distractibility is widely reported in tasks requiring selective attention. Several groups within the Western population are more easily distracted by task-irrelevant information than healthy young Western adults; these include typically developing young children [1], children with Attention Deficit Hyperactivity Disorder [2], the elderly [3], [4], and schizophrenic patients [5]. Such distractibility in selective attention tasks is important and related to everyday absent-mindedness and failures of attention [6], [7]. However, no group has so far been demonstrated to be less easily distracted by task-irrelevant information than healthy young Western adults.

In the present study, we investigate differences in distractibility in a cross-cultural comparison between Westerners and a non-Western population, namely the Himba, for whom we make the novel prediction that they may be better than young adults from the Western population at resisting distraction. It is possible to give a historical basis for our prediction. In the 18^th^ century, the idea that “primitive peoples” direct their attention to a small number of objects was widely circulated [8]. In more recent times, Jung [9] talked of the “astonishing concentration” of such peoples for things that interested them. None of these claims were backed up with experimental evidence. Here we provide the first empirical evidence of a population with an ability to concentrate on a visual task that is greater than it is in Westerners.

The Himba are a remote semi-nomadic culture in northern Namibia. We have recently reported evidence that, compared to Westerners, the Himba show a greater tendency to process the local features of an image rather than the global structure. Unlike Westerners, the Himba match compound stimuli based on their local, rather than global, similarity [10]. Also, their size judgments of target circles were little affected by the size of surrounding circles, suggesting that the Himba experience considerably less Ebbinghaus illusion than Western controls and as a result produced more accurate size judgments [11]. We interpreted these findings as evidence that the Himba have a local bias in visual processing that is stronger than that observed in Westerners.

In Experiment 1, we used a flanker task [12] to investigate whether attentional selectivity is better in the Himba than in Westerners. In the flanker task, irrelevant distractors are presented alongside a relevant to-be-attended target. If the distractors are imperfectly ignored, there will be a target-distractor compatibility effect (i.e., slower and less accurate performance when the distractor is associated with another response to the one required by the current target compared to when target and distractor are both associated with the same response). In the current study, participants were asked to respond to the direction of a target arrow (left or right), while ignoring two peripheral arrows. Relative to the target arrow, these distractor arrows could have either the same direction (compatible distractor condition) or the opposite direction (incompatible distractor condition). The extent to which the distractors were processed was determined by computing the difference in response latency between compatible and incompatible distractor conditions. If the local bias in the Himba is founded on a greater facility than in Westerners for attending selectively, then their compatibility effects should be reduced compared to those of Westerners.

We had two hypotheses about the origin of possible differences in distractibility between Himba and Westerners. Our first, ‘perceptual processing’, hypothesis was that reduced distractibility in the Himba could derive from a reduced capacity to process perceptually demanding displays. Our second, ‘attentional control’, hypothesis was that reduced distractibility in the Himba could derive from superior attentional control, allowing them to concentrate on the task in hand and thus to resist distraction from irrelevant information. We compared the two hypotheses by manipulating the level of perceptual load of the displays. Previous work shows that the extent to which irrelevant distractors are processed depends critically on the extent of the perceptual processing demands of the visual task [13]; increasing the level of perceptual load by presenting a target among various non-target items, rather than on its own, leads to significantly reduced distraction from an irrelevant distractor. Differences in perceptual processing capacity between Himba and Westerners should lead to interference being particularly reduced at intermediate levels of perceptual load in the Himba compared to Westerners [3]. In conditions of both low and high perceptual load, distractor effects may be similar in the Himba and Westerners. Under low perceptual load, both groups are predicted to have sufficient processing capacity to process the distractors and have sizeable interference effects. Under high perceptual load, for both groups, processing capacity is predicted to be exhausted, thereby preventing processing of the distractors and eliminating interference. Such a pattern of interference effects has indeed been reported for older Western participants, and interpreted to reflect reduced perceptual processing capacity [3]. However, if differences in perceptual capacity are not at the root of the cultural difference and our attentional control hypothesis is correct, distraction effects should be reduced in the Himba, compared to Westerners, even at the lowest level of perceptual load.

In sum, the aim of the current study was to explore the role of distractibility in the local bias found in the visual processing of a remote culture. We compared the ability to selectively attend to target information and ignore distracting information between Westerners and a remote culture. In Experiment 1, the Himba were less distracted by irrelevant flanker arrows, even at a low level of perceptual load. To establish that this effect could not be explained in terms of cultural differences in familiarity with the task or stimuli, we conducted Experiment 2 in which we presented distracting stimuli with a sudden onset. The capture of attention produced by such stimuli is particularly difficult to guard against, yet this study showed again that the Himba are less distractible than Westerners. Our results show that the Himba are significantly less distracted by task-irrelevant visual information than are Westerners, suggesting that their local processing bias may derive from a superior attentional control for task-relevant information.

## Experiment 1

### Methods

#### Ethics Statement

The study was approved by the College Ethics Committee at Goldsmiths, University of London. This study involves work with Western adults and also those from a non-literate indigenous group (Himba) with issues relating to vulnerable populations. Experimental work is planned from Goldsmiths Psychology Department where there are ethical procedures in place satisfying British Psychological Society (BPS) and UK Research Council (ESRC) requirements. Recruitment of Himba participants was through local recognised guides in cooperation with village chiefs. Himba participants always volunteer, and are never approached, to take part. Before the test, each participant was informed orally that they were free not to participate and that they could withdraw from the test at any time. Furthermore, the investigators would terminate the testing session if ever the participant showed signs of distress. In implementing the proposed research, we conformed to the Helsinki Declaration (in particular Article 24) in its latest version.

#### Participants

Participants were 55 (26 men and 29 women) adult monolingual Himba from an isolated region in Northern Namibia (mean estimated age: 25 years, 8 months; range: 17–45 years). Thirty-five further participants (13 men and 22 women) were native English speakers (mean age: 21 years, 9 months; range: 18–37 years). The English participants received course credits. The Himba were rewarded in kind; they received two gifts (1 kg of flour and 1 kg of sugar) at the end of the test. 

No cases of abnormal vision were reported.

#### Stimuli

The experiment was run using E-prime software [14]. Stimuli were presented on a 20-in screen at a viewing distance of 70 cm. To optimize timing accuracy we used a CRT monitor for both Himba and Western participants.

**Figure 1:**
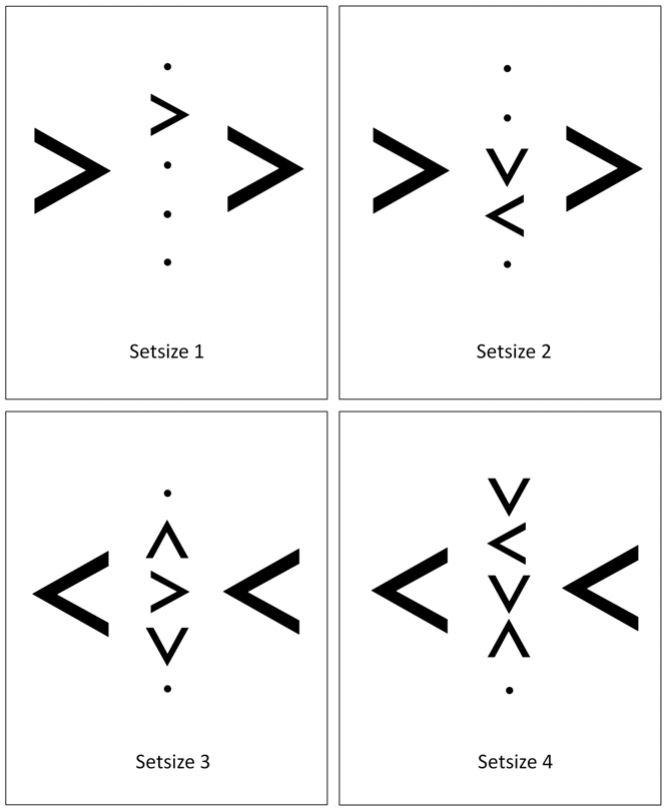
Examples of the stimulus displays for each condition of perceptual load in **Experiment 1** **.** Top left and bottom right panels are compatible displays, top right and bottom left panels are incompatible displays. Images not to scale.

See Figure 1 for example stimuli. Each stimulus display consisted of black arrow stimuli presented on a white ground. A target arrow subtending a visual angle of 1.40° horizontally and 1.75° vertically and pointing either to the left or the right was equally likely to appear in each of three possible positions, arranged either at fixation or 2.40° vertically above or below it. To manipulate perceptual load, the left or right pointing target arrow could either appear on its own (set size 1), or with one (set size 2), two (set size 3), or three (set size 4) additional non-targets. Non-targets were the same size as target arrows but pointed either up or down and could occupy any one of the three possible target positions not occupied on any trial by the target arrow, as well as two additional positions above and below these positions. Non-target and target arrows always occupied adjacent positions. Each display also contained two left or two right pointing distractor arrows, subtending a visual angle of 2.80° horizontally and 3.50° vertically. Distractor arrows were presented along the horizontal midline of the screen and at a distance of 4.8° (centre to centre) from the central target location. On half of the trials, the direction of the distractor arrows was the same as that of the target (compatible trials) whereas, on the other half of the trials, the direction of the distractor arrows was opposite to the target direction (incompatible trials).

#### Procedure

All participants were instructed to make a speeded key press to indicate the orientation of a target arrow, by pressing the left button on a two-button response box with their left hand for left target arrows, and the right button with their right hand for right target arrows. For the Himba participants, these instructions were given with the help of an interpreter who was naive to the purpose of the study. Our study is one of the first to measure response latencies in the Himba (also see [15]). To maximise the possibility that the Himba would be able to do the task and produce interpretable data, we first tested groups of Himba and Western participants in a version of the experiment in which the stimulus was displayed until a response was recorded (long exposure condition). When it appeared that the Himba participants readily understood the instructions, and no participants had to be excluded because they were outliers in terms of latency or accuracy, we tested additional groups of Himba and Western participants with a brief exposure duration of 220 ms. The long exposure condition included 23 Himba (11 men and 12 women; mean estimated age, 26 years; range, 17–45 years) and 13 Westerners (four men and nine women; mean age, 22 years and 11 months; range, 18–37 years). The brief exposure condition included 32 Himba (15 men and 17 women; mean estimated age, 25 years and 5 months; range, 17–40 years) and 22 Westerners (nine men and 13 women; mean age, 21 years and 1 month; range, 18–26 years).

Each trial started with a central fixation cross, displayed for 1400 ms, followed by the stimulus display presented until a response was recorded (long exposure condition), or for 220 ms, and followed by a blank screen until a response was recorded (brief exposure condition). There was no feedback for incorrect responses. For each perceptual load condition, all combinations of target orientation (left, right), target location (three positions), distractor orientation (left, right) were used to create 12 unique trials. These were randomly presented ten times in each experimental block of 120 trials. Thus, all within-subjects factors were manipulated within blocks apart from perceptual load which was varied between blocks. The order of blocks was varied between participants using a Latin square. Each participant first completed four practice blocks, one for each level of perceptual load, each consisting of 24 randomly selected trials from each load condition, followed by the four experimental blocks. At the end of each perceptual load block participants were allowed a short break. The Himba were tested individually in a dimly lit tent, the Westerners in a dimly lit testing cubicle.

### Results

**Figure 2:**
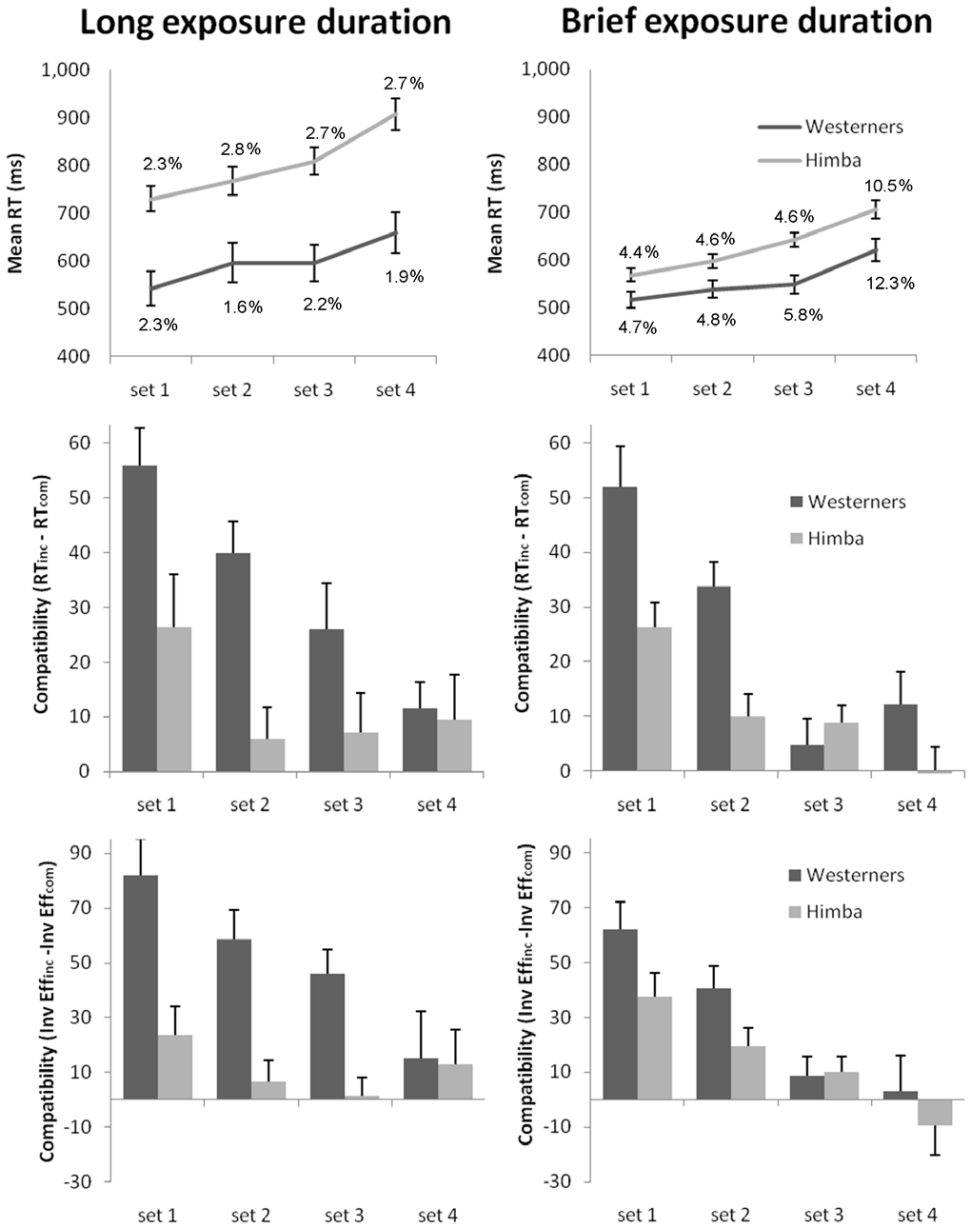
Results for **Experiment 1** **.** Left panels present the data from the long exposure duration condition, right panels present the data from the brief exposure duration condition. (A) Overall mean reaction time and error rate for Westerners and Himba as a function of set size and exposure duration. Error bars represent standard error. Percentage values are overall error rates. (B) Mean distractor compatibility effects (reaction time) for Westerners and Himba as a function of set size and exposure duration. *, p<.05; **, p<.01; ***, p<.001. Error bars represent standard error. (C) Mean distractor compatibility effects (inverse efficiency scores) for Westerners and Himba as a function of set size and exposure duration. *, p<.05; **, p<.01; ***, p<.001. Error bars represent standard error.

Figure 2A presents the mean correct reaction time and error rates for Himba and Western participants in the long and brief exposure durations as a function of set size. Figure 2B and Table 1 present the mean distractor compatibility effects. In the brief exposure condition, there were signs of a speed-accuracy trade-off, with slower and more accurate responses in the Himba, and faster and less accurate responses in the Westerners (right hand panel of Figure 2A). To exclude the effect of any speed-accuracy trade-offs from our analysis, we computed the inverse efficiency for each condition for all participants, by dividing mean correct reaction time by the proportion of correct responses for that condition [16], [17]. We computed compatibility effects as a function of perceptual load for each participant, by subtracting the compatible inverse efficiency from the incompatible inverse efficiency at each set size (see Figure 2C). These compatibility effects were entered in a 2×2×4 mixed Analysis of Variance (ANOVA), with culture (Western, Himba) and exposure duration (long, brief) as the between subjects factors, and perceptual load (set size 1, set size 2, set size 3, set size 4) as a within-subject factor. There was a main effect of load, F(2.6,220) = 18.04, MSe = 2087.08, p<.001, η*_p_* ^2^ = .173 (Greenhouse-Geisser corrected): distractor compatibility effects were reduced in magnitude with increases in set size (set size 1, M = 51 ms; set size 2, M = 31 ms; set size 3, M = 16 ms; set size 4, M = 5 ms); this replicates previous findings of perceptual load on distractor interference [16]. There was a marginally significant interaction between culture and exposure duration, F(1,86) = 3.95, MSe = 3252.43, p = .05, η*_p_* ^2^ = .044. In Westerners, compatibility effects were greater in the long compared to the brief presentation condition (M = 50 ms and M = 29 ms, respectively). No such difference occurred in the Himba compatibility effects (M = 11 ms and M = 14 ms, for long and brief durations, respectively). Crucially, there was a significant effect of culture, F(1,86) = 17.87, MSe = 3252.43, p<.001, η*_p_* ^2^ = .172: the overall compatibility effect was substantially greater in the Westerners (M = 39 ms) than the Himba (M = 13 ms). No other effects were significant. Similar separate analyses on latencies and error rates (rather than inverse efficiency scores) produced the same key result of significantly reduced compatibility effects in the Himba compared to Westerners.

**Table 1:** Compatibility effects in accuracy rates (percent) as a function of set size, exposure duration, and culture; values in brackets represent standard error.

		Set Size			
		Set 1	Set 2	Set 3	Set 4
Long Exposure Duration	Westerners (n = 13)	3.5 (1.6)	2.4 (1.3)	2.6 (1.1)	.5 (.4)
	Himba (n = 23)	−.4 (.4)	.1 (.6)	−.6 (.6)	.3 (.6)
Brief Exposure Duration	Westerners (n = 22)	1.4 (1.1)	.9 (.8)	.8 (.7)	−.8 (1.1)
	Himba (n = 32)	2.0 (.9)	1.3 (.6)	.1 (.5)	−.1 (.9)

We checked that the effects from the inverse efficiency analysis would be replicated in conventional separate analyses on the reaction times and error rates. First, compatibility effects in the latencies were analysed in the same 2×2×4 ANOVA as used for the main analysis. The key finding was again of reduced distractibility in the Himba compared to Westerners, as shown by a significant main effect of culture, F(1,86) = 21.15, MSe = 1220.84, p<.001, η*_p_* ^2^ = .197. Whereas the Westerners showed an overall compatibility effect of 29 ms, the overall compatibility effect was much reduced in the Himba, M = 12 ms. Second, the same three-way ANOVA was used to analyse the mean error rates. Again, we found that overall distractibility was significantly reduced in the Himba (M = 0.33) compared to the Westerners (M = 1.40), as shown by a main effect of culture, F(1,86) = 4.32, MSe = 21.60, p<.05, η*_p_* ^2^ = .048. These analyses on latencies and error rates produced additional interactions that were eliminated when these measures were combined in the inverse efficiency scores used in the main analysis.

Overall, the Himba responded slower (M = 716 ms) than the Westerners (M = 577 ms, F(1,86) = 33.46, MSe = 93319.09, p<.001, η*_p_* ^2^ = .280). In order to ensure that any difference in distractibility between Himba and Westerners was independent of the effect of overall latency, we also performed an analysis on the latencies (rather than the inverse efficiency scores) from the faster Himba and slower Westerners. We performed a matched-pairs analysis, selecting pairs of participants from each exposure condition, one from each group, with near identical reaction times. A total of 19 pairs were selected. In these pairs, overall latencies were the same in the Himba (M = 616) and the Westerners (M = 617, F<1). The compatibility effects for these matched pairs were entered into the same 2×2×4 ANOVA as above. In the matched pairs analysis, the key effect of culture was again significant, F(1,34) = 7.02, MSe = 952.25, p<.02, η*_p_* ^2^ = .171. The overall compatibility effect was 24 ms in the Westerners, compared to 9 ms in the Himba.

Finally, as gender differences have been reported in terms of local/global visual processing [18], we wanted to ensure that none of the differences in distractibility between cultures could be attributed to a difference in the gender distribution between our Himba (47% male) and Western (37% male) samples. There was no main effect of gender on inverse efficiency compatibility effects (p>.29), and neither did gender interact with any of the other factors (all ps>.19).

### Discussion

Of the two hypotheses under test (perceptual processing capacity vs. attentional control), it seems unlikely that our data can be explained by a reduced capacity to process perceptual information. If this were the case, then the Himba would have been expected to be influenced by the distractors to a similar extent as Westerners when perceptual load was negligible (set 1), but not as set size increased and their perceptual capacity was exceeded. Instead, the cultural difference in distractibility was independent of perceptual load, and the Himba were already considerably less distractible than Westerners at the lowest level of load (see Figure 2B and 2C). The finding that the Himba are less distractible, regardless of the perceptual load of the displays, suggests that this reduced distractibility results from a superior ability to allocate attention to the task in hand.

An obvious concern is whether the reduced interference effects in the Himba can be explained by differences between Westerners and Himba in their familiarity with the stimuli. The extant literature would suggest they cannot. Considering familiarity with respect to effects of practice within an experimental session, it has been found that distractor effects become smaller with increasing practice across ten blocks of trials [19]. Moreover, when the identity of the distractors was changed halfway through the experiment, the new items again produced strong interference effects, showing that stimulus novelty is associated with greater, rather than reduced distractibility. Considering familiarity with respect to task expertise, under low perceptual load, experts were as distractible [20], [21] or even less distractible [22], than non-experts. There is some evidence that distractor effects can increase with expertise, but this is exclusively under high perceptual load [20], [21] (see [23] for evidence that expertise can also be associated with reduced distractibility under high perceptual load). In sum, the evidence on expertise and attention does not point to a clear association between these factors. Nonetheless, we wanted to establish in a further study that the Himba would also be less distractible in a task in which familiarity and task experience are less likely to have an effect; such a task is attentional capture by motion singletons.

Attentional capture refers to the disruption of visual search by the presence of a salient individual item (singleton) in the search array, suggesting that attention is captured by the singleton and shifted towards its position (see [24] for a recent review of the attentional capture literature). Capture of attention by distractor singletons, and especially capture by sudden onset and motion, is widely regarded as something that is particularly difficult to guard against, as it is in part exogenously driven, and less influenced by top-down control than for example the flanker effects we used in Experiment 1 [24]–[27]. There is some debate as to whether motion is as effective in capturing attention as are sudden onsets. Importantly, a moving singleton distractor that remains in the same overall position (e.g., the singleton rotates around its center) does not always capture attention [28], [29]. In contrast, when the motion involves a movement of the singleton to a new location, capture tends to be robust [30]. In Experiment 2, we therefore manipulated motion by changing the location of the singleton distractor. In the Western data, such motion singletons indeed produced robust attentional capture. Given its exogenous nature, capture by onset or motion should be less sensitive to stimulus familiarity and task experience. In Experiment 2, in order to further minimise group differences in familiarity with the stimuli, we also included a stimulus category with which the Himba are arguably more familiar than the Westerners, namely pictures of cows.

There is another, related, reason why we wanted to add a comparison of Western and Himba motion capture effects. The key conclusion from Experiment 1 was that the Himba were less distracted than the Westerners, even under the lowest level of perceptual load. We thus assume that the subjective level of perceptual load was the same in the two groups, whereas it may be that the perceptual processing demands of the same displays were greater for the Himba, for whom the displays were unfamiliar. Although there is evidence that attentional capture by colour can be reduced when the perceptual load of the display is high [31], no such evidence exists for motion capture. In Experiment 2, we therefore assumed that a reduction in the motion capture effect in the Himba compared to the Westerners could not be attributed to differences in their subjective levels of perceptual load of the displays.

## Experiment 2

### Methods

#### Participants

Experiment 2 included 28 Himba participants (11 men and 17 women; mean estimated age: 25 years, 5 months; range: 16–42 years) and 25 native English speakers (10 men and 15 women; mean age: 24 years, 4 months; range: 18–40 years), none of whom had participated in Experiment 1. None of the Himba participants had made more than three visits to the nearest regional town, Opuwo. All participants were rewarded as in Experiment 1. No cases of abnormal vision were reported.

#### Stimuli

**Figure 3:**
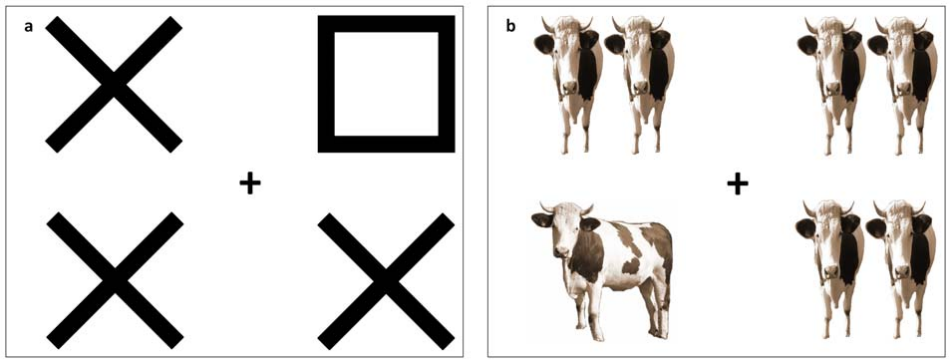
Examples of the shape and cow stimulus displays. In each display, the unique item was the target (square in the shape example, left pointing cow in the cow example). On half the trials, one of the remaining items (the singleton) moved back and forth. Images not to scale.

See Figure 3 for example stimuli. Each stimulus display consisted of four images, arranged in a rectangle subtending 10.2° horizontally and 8.6° vertically, and presented centred on a white background. Images were squares, circles and crosses (each subtending 3.9° square) in the shape condition and photographs of a cow or pair of cows (each subtending 3.9° square) in the cow condition. In the shape condition, the display consisted of a square or a circle (the target) presented among three crosses (the non-targets). In the cow condition, the display consisted of one cow pointing left or right (the target) and three pairs of cows facing forward (the non-targets).

#### Procedure

In the shape condition, participants searched for a target shape, and were instructed to use their left or right hand to make a speeded key press to indicate if it was a square or a circle, by pressing the left button for a square and the right button for a circle on a two-button response box. In the cow condition, participants searched for the target cow, and pressed the left button if it was facing leftwards and the right button if it was facing rightwards. A central fixation cross was displayed throughout the experiment. 500 ms after the beginning of each trial, the stimulus display was presented for 200 ms. On half of the trials in both the shape and the cow condition, 50 ms after stimulus onset, one of the non-targets (the singleton) shifted 3.2° further to the left (for non-targets on the left of the array) or to the right (for non-targets on the right of the array) staying in the new position for 100 ms, after which it shifted back to its original position for another 50 ms. Thus, a singleton was created by the outward and inward movement. On the other half of trials, the singleton-absent trials, the target and non-targets were presented together for 200 ms without any changes in their locations.

A new trial started after a response was recorded or after 5000 ms had elapsed. For each of the four target positions in the shape condition, all combinations of target shape (square or circle) and singleton position (three positions) were used to create 24 unique shape trials. Similarly for each of the four target positions in the cow condition, all combinations of target orientation (left or right) and singleton position (three positions) were used to create 24 unique cow trials. These were randomly presented twice (each experimental condition thus consisted of 48 trials). Shape and cow conditions were presented in separate blocks and were performed in counterbalanced order across participants. Before each condition, participants first completed a practice block. The practice block terminated after participants completed six consecutive practice trials with no more than one mistake.

### Results

**Figure 4:**
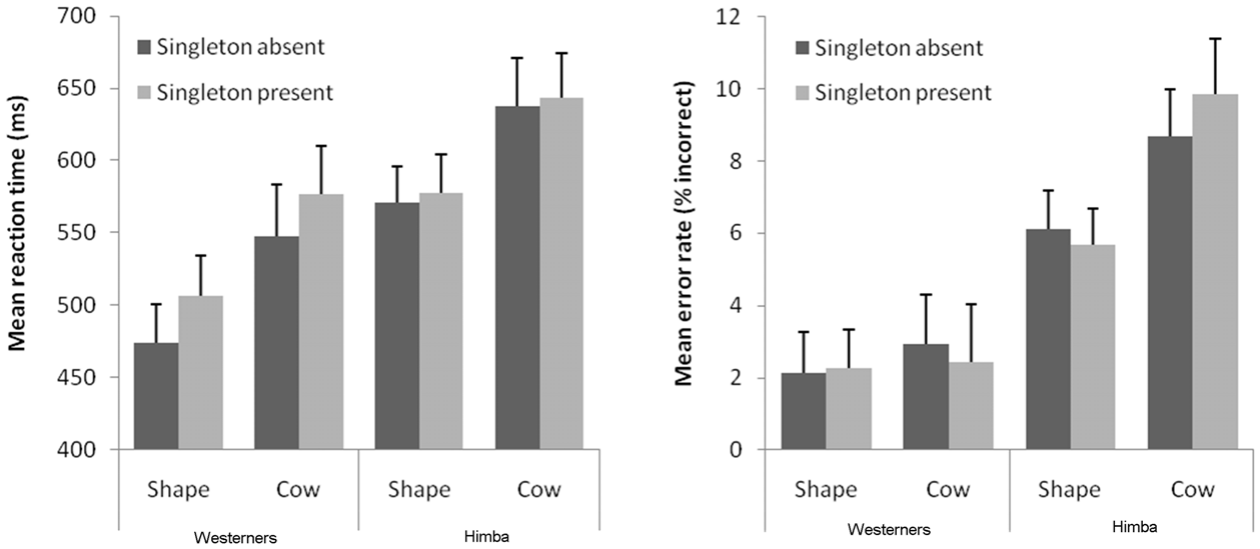
Results for **Experiment 2** **.** Reaction times (left panel) and error rates (right panel) are presented for Westerners and Himba as a function of singleton type and presence. Error bars represent standard error.

Figure 4 presents the mean correct reaction time and error rates for Himba and Western participants as a function of singleton presence and singleton type. In the error rates in Experiment 2, there was only a main effect of culture, and no other significant effects. There was therefore no risk of any speed-accuracy trade-off in the data, and we therefore analysed the reaction times and error rates in the conventional way. Reaction times were entered in a 2×2×2 mixed ANOVA, with culture (Western, Himba) as a between-subjects factor, and singleton presence (present, absent) and stimulus category (shape, cow) as within-subjects factors. There was a main effect of culture, F(1,51) = 4.64, MSe = 75063.63, p<.05, η*_p_* ^2^ = .083: reaction times were faster in Westerners (M = 526 ms) compared to the Himba (M = 607 ms). The main effect of singleton presence was also significant, F(1,51) = 11.92, MSe = 1541.79, p<.01, η*_p_* ^2^ = .189). Reaction times were slower in singleton present (M = 576 ms) compared to singleton absent conditions (M = 557 ms). The main effect of stimulus category was also significant, F(1,51) = 14.83, MSe = 17177.99, p<.001, η*_p_* ^2^ = .225). Reaction times were slower in cow (M = 601 ms) compared to shape conditions (M = 532 ms). Importantly, the only significant interaction was between culture and singleton presence, F(1,51) = 5.07, MSe = 1541.79, p<.05, η*_p_* ^2^ = .090: the singleton capture effect was substantially greater in the Westerners (M = 31 ms) than the Himba (M = 7 ms). In fact, whereas the motion singleton reliably captured attention in Westerners in both the shape condition (t(24) = 2.64, SEM = 12.32, p<.025, two-tailed) and the cow condition (t(24) = 2.82, SEM = 10.30, p<.01, two-tailed), the singleton capture effect was not different from zero in the Himba in either the shape condition (t(27) = .65, p>.5) or the cow condition (t(27) = .49, p>.6). No other interactions were significant (all Fs<1).

Mean error rates were analysed in a similar 2×2×2 ANOVA. Overall error rates were low (5%), and the only significant effect was a main effect of culture, F(1,51) = 15.39, MSe = 90.68, p<.001, η*_p_* ^2^ = .232. Error rates were higher in the Himba (M = 7.58%) than in Westerners (M = 2.44%). The main effect of stimulus category was marginally significant, F(1,51) = 3.92, MSe = 50.12, p = .053, η*_p_* ^2^ = .071, with more errors in the cow condition (M = 5.97) than in the shape condition (M = 4.05). Neither shapes nor cows produced significant motion capture in the error rates in either Westerners or Himba (all ps>.4).

As in Experiment 1, the Himba responded significantly slower than the Westerners in Experiment 2. In order to ensure that the difference in attentional capture between Himba and Westerners was independent of the effect of overall latency, we again performed an analysis on the latencies from the faster Himba and slower Westerners. We again performed a matched-pairs analysis, selecting 17 pairs of participants, one from each group, with near identical reaction times. In this analysis, overall latencies were the same in the Himba (M = 574) and the Westerners (M = 577, F<1), yet the key interaction between culture and singleton capture was again significant, F(1,32) = 4.71, MSe = 1953.55, p<.05, η*_p_* ^2^ = .128. The overall capture effect was 30 ms in the Westerners, compared to −3 ms in the Himba.

### Discussion

The results of Experiment 2 are straightforward. Motion singletons produced reliably more attentional capture in the Westerners compared to the Himba, to the extent that capture effects in the Himba were no different from zero. For a motion singleton to fail to produce any capture is a strong finding. Previous work suggests that motion capture effects are especially robust compared to capture by colour or shape singletons and that they may reflect exogenous capture of attention [26], [27], [30]. For our purposes, the finding clearly suggests that differences in familiarity are unlikely to explain the reduction in distractibility we observe in the Himba. The finding also suggests that the cultural difference in distractibility observed in Experiment 1 is unlikely to be due to a difference in the subjective level of perceptual load. Finally, the total absence of a three-way interaction (F = .008) suggests again that stimulus familiarity has little influence on these effects: Himba showed greatly reduced attentional capture effects compared to Westerners with both shapes (presumably more familiar to Westerners) and cows (presumably more familiar to the Himba).

## Discussion

The Himba ability to identify a target was significantly less affected by distracting visual information compared to Westerners. Finding a population with better attentional selectivity than healthy Western controls is especially remarkable given that the ability to stay focused on a task by limiting the adverse effects of interference is key to many activities in Western culture, including cognitive tasks such as reading and problem solving, and visuo-motor tasks such as driving. Many of these tasks are either unknown to the Himba or not relevant to their daily lives, suggesting that cultural differences in the relevance of, or experience with, such tasks cannot form the basis for their superior attentional selectivity.

Our findings cannot be explained in terms of methodological issues regarding cultural differences in either response latencies or comprehension of task instructions. In both experiments, the difference in distractibility between the Himba and Westerners was observed both with and without a cultural difference in overall latency; this clearly suggests that slower Himba performance could not explain our findings. Indeed, the fact that the reduction in distractibility in the Himba was also observed in the context of their slower overall performance is a strong finding, as it makes it impossible to explain these findings in terms of scaling: the longer latencies in the Himba compared to the Westerners were associated with smaller, rather than larger, compatibility effects. In other words, general task performance in terms of speed of responding was better in Westerners compared to the Himba, but the Himba outperformed the Westerners on one specific aspect of the task, namely the ability to prevent processing of distracting information. One also cannot look to a misunderstanding of the task to explain the data. In Experiment 1, the overall pattern of the compatibility and perceptual load effects was very similar between cultures, with both groups showing poorer performance on incompatible versus compatible trials, and as a function of increasing set size. Similarly in Experiment 2, the direction of capture effects was similar across cultures. The Himba performance was simply less affected by both the distractor arrows and the motion singletons.

It is not our major concern to offer a causal explanation for the local bias/non-distractibility of the Himba but we do make some observations. First, we emphasise that we do not wish to align the Himba to groups within Western culture who also show an enhanced local bias such as patients with Autistic Spectrum Disorder [32], Western males [18] (but see [33] for evidence of no gender difference in local bias), and young children [34]. Although these groups are like the Himba in having a tendency towards local processing, only the Himba express a greater facility for attentional selectivity (e.g. [35], [36]). With the Himba we have a rare demonstration of a specific population showing reduced distractibility compared to Western control participants, that is in the opposite direction to the finding for groups such as children [1], children with Attention Deficit Hyperactivity Disorder [2], the elderly [3], [4], and schizophrenic patients [5] who have greater distractibility than Western controls.

Our second observation is that our findings do not support widely held views concerning the basis of cultural differences in local/global processing. The Himba local bias is opposite to findings in Japanese observers, who show a global perceptual bias relative to Westerners [37], [38]. The difference in local/global processing between Japanese and Western participants is often interpreted in terms of differences in social organization [37], such that the greater global bias in Japanese observers relative to Westerners reflects the more local processing associated with Western individualistic society, and more global processing with Japanese collectivist society. However, there is no evidence that the Himba society is more individualistic than Western society, so the social organization account fails to explain the Himba local bias [39].

Our third observation is that our data are compatible with the visual-clutter account of local/global bias [38], which explains a local bias as an adaptation to low-clutter environments. The Himba visual environment consists mostly of open rural landscape, and is much less cluttered than the urban London environment in which our Western controls lived. In a non-cluttered visual environment such as that of the Himba, attention can be readily directed to the relevant information as targets are easily distinguished from the background. It has been argued that a local processing style is more suited to such environments; in contrast, the ambiguity of a cluttered visual environment such as that in urban London would promote a global bias, as the background often needs to be inspected in order to successfully select the target [38], [40]. The greater distractor effects we found in our Western group may therefore be a result of this tendency for global processing. It could therefore follow that distractibility may be an indirect consequence of urbanisation. In any case, the Himba and presumably all other remote groups offer the opportunity to study the factors that explain individual differences in distractibility, and can lead to improved attentional selectivity.
